# NMNAT1 Is a Survival Factor in Actinomycin D-Induced Osteosarcoma Cell Death

**DOI:** 10.3390/ijms22168869

**Published:** 2021-08-18

**Authors:** Alexandra Kiss, Csaba Csikos, Zsolt Regdon, Zsuzsanna Polgár, László Virág, Csaba Hegedűs

**Affiliations:** 1 Department of Medical Chemistry, Faculty of Medicine, University of Debrecen, H-4032 Debrecen, Hungary; kissalexandra@med.unideb.hu (A.K.); csikosc1997@gmail.com (C.C.); regdike1988@gmail.com (Z.R.); polgar.zsuzsanna@med.unideb.hu (Z.P.); 2 Doctoral School of Molecular Medicine, University of Debrecen, H-4032 Debrecen, Hungary; 3 MTA-DE Cell Biology and Signaling Research Group, H-4032 Debrecen, Hungary

**Keywords:** NAD^+^, NMNAT1, actinomycin D, chemotherapy, apoptosis, PARP1, SIRT1, osteosarcoma, cancer, high throughput screening

## Abstract

Osteosarcoma is a frequent and extremely aggressive type of pediatric cancer. New therapeutic approaches are needed to improve the overall survival of osteosarcoma patients. Our previous results suggest that NMNAT1, a key enzyme in nuclear NAD^+^ synthesis, facilitates the survival of cisplatin-treated osteosarcoma cells. A high-throughput cytotoxicity screening was performed to identify novel pathways or compounds linked to the cancer-promoting role of NMNAT1. Nine compounds caused higher toxicity in the NMNAT1 KO U2OS cells compared to their wild type counterparts, and actinomycin D (ActD) was the most potent. ActD-treatment of NMNAT1 KO cells increased caspase activity and secondary necrosis. The reduced NAD^+^ content in NMNAT1 KO cells was further decreased by ActD, which partially inhibited NAD^+^-dependent enzymes, including the DNA nick sensor enzyme PARP1 and the NAD^+^-dependent deacetylase SIRT1. Impaired PARP1 activity increased DNA damage in ActD-treated NMNAT1 knockout cells, while SIRT1 impairment increased acetylation of the p53 protein, causing the upregulation of pro-apoptotic proteins (NOXA, BAX). Proliferation was decreased through both PARP- and SIRT-dependent pathways. On the one hand, PARP inhibitors sensitized wild type but not NMNAT1 KO cells to ActD-induced anti-clonogenic effects; on the other hand, over-acetylated p53 induced the expression of the anti-proliferative p21 protein leading to cell cycle arrest. Based on our results, NMNAT1 acts as a survival factor in ActD-treated osteosarcoma cells. By inhibiting both PARP1- and SIRT1-dependent cellular pathways, NMNAT1 inhibition can be a promising new tool in osteosarcoma chemotherapy.

## 1. Introduction

Osteosarcoma is the most common primary bone cancer [1]. About 3–4 out of one million people develop osteosarcoma each year [2]. Osteosarcoma is more common in adolescents aged 15–19 years, where it accounts for >10% of all solid tumors, and in people >70 years old. The average age at diagnosis of this tumor type is 15 years. Men are affected slightly more often. Osteosarcoma is an aggressive, fast-growing tumor that can lead to death within a year if left untreated [2]. In addition to surgery, adjuvant and neoadjuvant combination chemotherapy is the primary osteosarcoma treatments. The most commonly used chemotherapeutic drugs in osteosarcoma patients include doxorubicin, cisplatin, and methotrexate [3]. However, despite significant advances in the development of cancer therapies in general, the prognosis of osteosarcoma has not improved in the last 30 years. Thus, further preclinical and clinical research is needed to find and validate new therapeutic targets for osteosarcoma.

Our previous study demonstrated that targeting nicotinamide adenine dinucleotide (NAD^+^) metabolism improves the efficacy of cisplatin and doxorubicin in osteosarcoma treatment [4]. NAD^+^ is a coenzyme in many redox reactions in normal tissues, and in cancer, NAD^+^ is required in metabolic pathways for survival and growth. Most notably, poly(ADP-ribose) polymerase(PARP), and SIRT enzymes represent major NAD^+^-consuming enzymes involved in the regulation of DNA repair, metabolism, gene expression regulation, etc. [5,6,7], which are closely linked to cancer cell functioning, survival, and death. NAD^+^ can be synthesized in three different pathways: the Preiss-Handler pathway, which produces NAD^+^ from nicotinic acid; the de novo synthesis pathway, which produces NAD^+^ from tryptophan; and the salvage pathway, which produces NAD^+^ from nicotinamide (NAM) [8]. Although the salvage pathway is considered a default NAD^+^ synthesis route, the tissue context determines which of these metabolic pathways are predominantly used for NAD^+^ production in cancer cells [9]. Nicotinamide mononucleotide adenosyltransferase (NMNAT) enzymes are uniquely positioned in the NAD^+^ synthesis routes and cannot be bypassed in any of the NAD^+^ synthesis pathways. NMNAT has three isoforms: NMNAT1 is localized to the nucleus, whereas NMNAT2 and NMNAT3 occur in the cytoplasm. In addition, NMNAT2 localizes to the Golgi and NMNAT3 to the mitochondria [10]. Of note, the expression of NMNAT isoforms depends on the cell type [10].

The nuclear isoform, NMNAT1, interacts directly with PARP1 at the site of DNA damage [11] and stimulates PARP1 activity. PARP1 is a DNA nick sensor enzyme that becomes activated upon sensing DNA breaks [12,13,14]. Activated PARP1 uses NAD^+^ to synthesize poly (ADP-ribose) (PAR) polymers covalently attached to PARP1 (automodification) or other nuclear proteins, such as histones (heteromodification). The binding of NMNAT1 to PARP1 is dependent on the automodification state of PARP1, as interaction of the two enzymes occurs through NMNAT1 binding to the PAR polymer [11]. In addition to PARPs, another major NAD^+^ consumer enzyme family is represented by SIRTs. Although some SIRTs can catalyze mono-ADP-ribosylation of proteins, SIRTs primarily function as NAD^+^-dependent deacetylases [11].

Targeting NAD^+^ synthesis may be a viable strategy for combatting cancer. In particular, nuclear NAD^+^ synthesis appears to be a valid target, considering how closely NAD^+^ synthesis is intertwined with PARP1-dependent DNA repair mechanisms. Indeed, high NMNAT1 expression is associated with shorter survival in cancer patients [15], supporting this hypothesis. In our previous work, we showed that inactivation of the NMNAT1 gene sensitizes osteosarcoma cells to the cytotoxic effects of cisplatin and doxorubicin [4]. Moreover, we demonstrated that the chemosensitizing effect of the NMNAT1 gene inactivation compromises PARP1 activation, PAR synthesis, and DNA damage repair. In our current study, we aimed to identify novel pathways synergizing with NMNAT1 in the promotion of osteosarcoma cell survival. By screening a small library of FDA-approved drugs, we identified actinomycin D (ActD) as a compound synergizing with NMNAT1 gene inactivation to cause osteosarcoma cell death.

## 2. Results

### 2.1. Actinomycin D Has Synergistic Cytotoxic Effects with the NMNAT1 Knockout (KO) Phenotype

**Figure 1:**
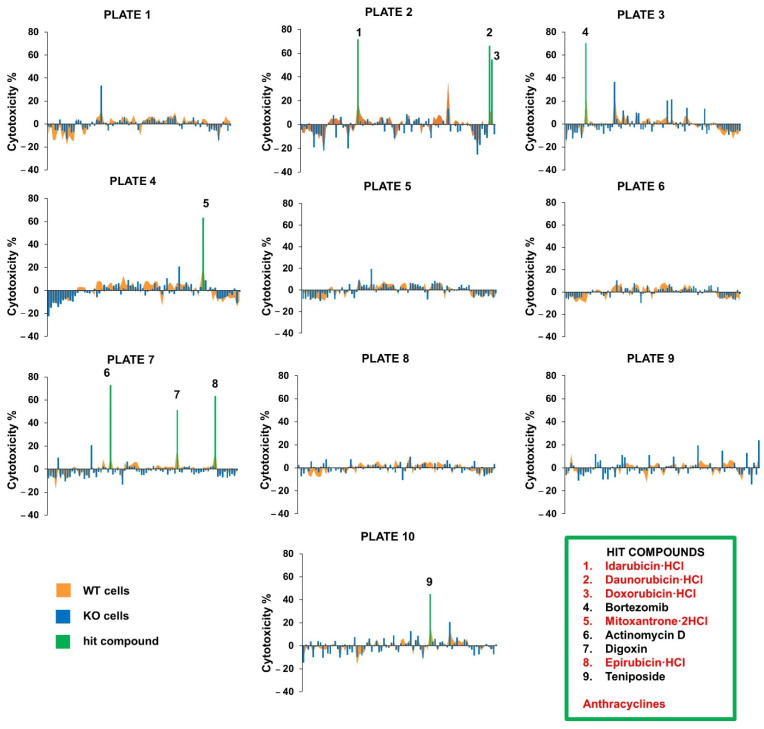
High throughput screening of 774 FDA-approved drugs. Drugs from the ScreenWell V2 library were applied to WT (orange chart) and NMNAT1 KO (blue columns) U2OS cells. Cells were treated with each drug for 24 h at 10 μM final concentration. Viabilities were determined with Calcein-AM viability assay in 10 microplates. Primary viability values were converted to the cytotoxicity percentage. The results of WT and NMNAT1 KO cells were merged and presented on graphs, marked by the plate number of the library plate (plates 1–10). Compounds showing at least 25% higher toxicity in the KO cells were selected as hit compounds (green columns). The names of the hit compounds are listed (1–9); the anthracyclines are shown in red color.

**Figure 2:**
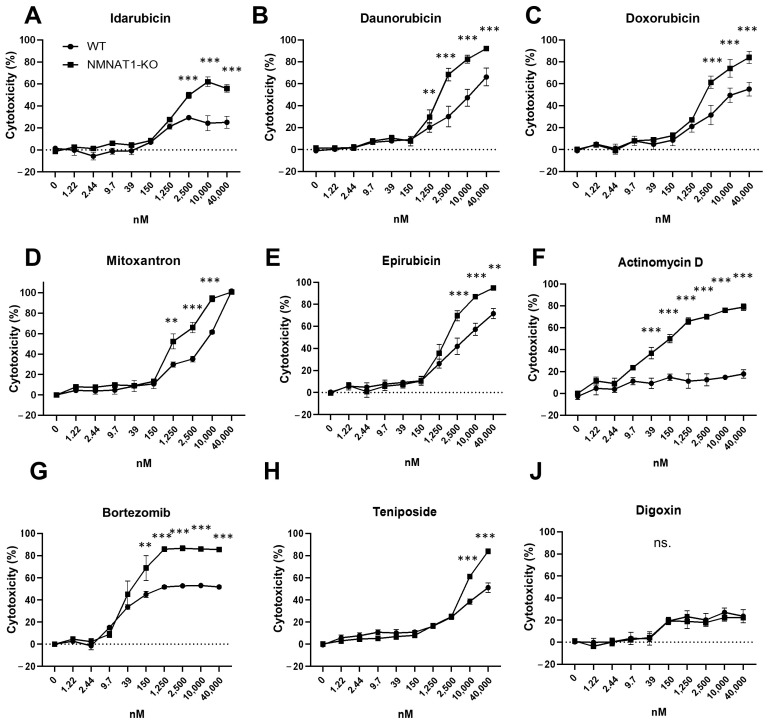
Validation of hit compounds. Nine compounds (idarubicin, (**A**); daunorubicin, (**B**); doxorubicin, (**C**); mitoxanthron, (**D**); epirubicin, (**E**); actinomycin (**D**,**F**); bortezomib, (**G**); teniposide, (**H**); digoxin, (**J**) were identified in the screen as hit compounds. Validation of the HTS assay was performed with manual treatment. (**A**) concentration series for each compound ranging from 1.22–40,000 nM was used and viability was measured using Calcein-AM assays. Data points, marked with asterisks, are significantly different between WT and KO cells (Sidak’s test; ** *p* < 0.01, *** *p* < 0.001, ns.: not significant). Data plotted are means ± SEM (*n* = 3).

To find compounds with synergistic cytotoxic effects with the NMNAT1 KO phenotype, a high throughput screen was performed with a small compound library containing 774 FDA-approved drugs. Wild type (WT) and NMNAT1 KO (KO) U2OS osteosarcoma cell lines were used for testing the cytotoxicity of the drugs in the NMNAT1 KO phenotype. Cytotoxicity was determined after 24 h of treatment in both cell lines. Compounds showing at least 25% higher toxicity on the NMNAT1 KO background were considered hits. Nine compounds, including bortezomib, actinomycin D, digoxin, teniposide, and five anthracyclines (idarubicin, daunorubicin, doxorubicin, mitoxantrone, and epirubicin), met the criteria (Figure 1). Hits were validated in the same model. The toxicity of the hit compounds was determined in 24-h viability assays using a concentration series (ranging between 1.22 nM–40 μM), including the 10 μM concentration used in the screen (Figure 2). Out of the nine compounds, the sensitizing effect on the NMNAT1 KO phenotype could be confirmed in eight cases. The effects of all five identified anthracyclines were verified (Figure 2A–E) when 1250 nM (daunorubicin, mitoxanthron), 2500 nM (idarubicin, doxorubicin, epirubicin), or higher concentrations were applied. ActD (Figure 2F), bortezomib (Figure 2G), and teniposide (Figure 2H) caused significantly higher cytotoxicities in the KO cell line compared to WT cells. Digoxin exhibited concentration-dependent cytotoxicity in both cell lines; however, the toxicity in the KO cell line was not significantly higher (Figure 2J). The most marked difference was observed with ActD; the lack of NMNAT1 caused a significant sensitizing effect starting from ~40 nM. Therefore, ActD was selected for further investigation.

### 2.2. Characterization of ActD Induced Cell Death

**Figure 3:**
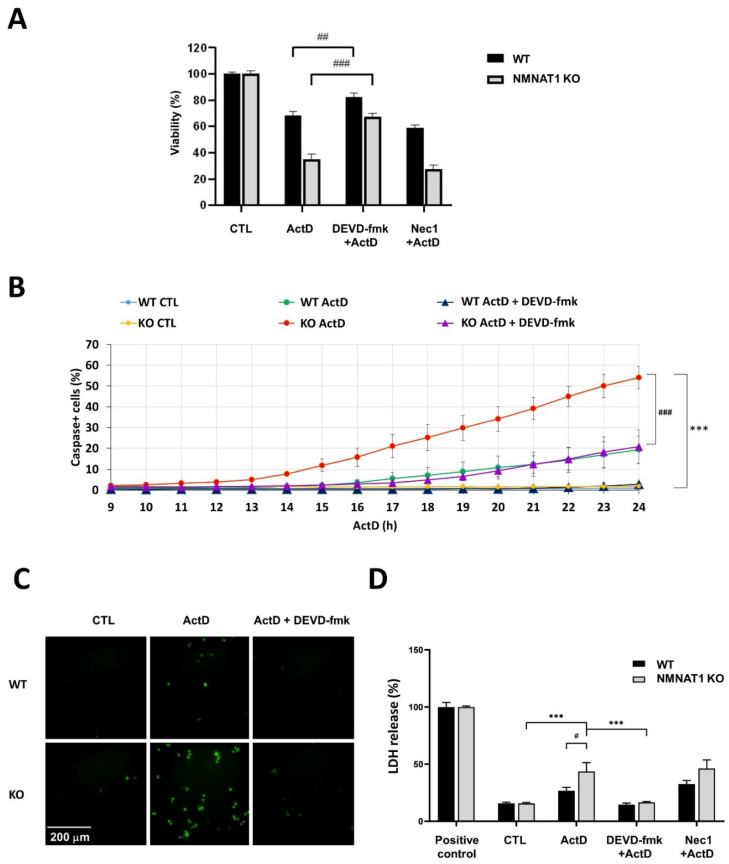
Actinomycin D (ActD) treatment results in apoptotic cell death in U2OS cells. The mechanism of ActD-induced osteosarcoma cell death was identified using viability assays (**A**) with inhibitors of apoptotic (DEVD-fmk; 50 μM) and necroptotic (Nec1; 1 μM) pathways. Bars marked with hash marks are significantly different from the same cell line treated only with ActD (Dunnett’s multiple comparisons; ## *p* < 0.01, ### *p* < 0.001). Apoptotic cell death was detected with a fluorescent caspase substrate (Cellevent green). In a kinetic caspase assay, the time course of caspase activity was determined 9–24 h after ActD treatment. The percentages of caspase-positive cells are presented (**B**). Curves marked with asterisks are significantly different from the control samples of the same cell line (Sidak’s multiple comparisons; *** *p* < 0.001). Curves marked with hash marks are significantly different from the same cell line treated only with ActD (Sidak’s multiple comparisons; ### *p* < 0.001). Representative images show caspase-positive cells after 24 h of ActD treatment (**C**). Lactate dehydrogenase (LDH) release, as a necrotic cell death parameter, was determined 24 h after ActD treatment (**D**). Bars marked with asterisks are significantly different from the control samples of the same cell line (Dunnett’s multiple comparisons; *** *p* < 0.001). Bars marked with hash marks are significantly different from the same cell line treated only with ActD (Dunnett’s multiple comparisons; # *p* < 0.05, ## *p* < 0.01, ### *p* < 0.001). Inhibitors were added 30 min before ActD treatment (**A**–**D**). Means ± SEM are shown on panels (**A**,**B**,**D**) (*n* = 3).

ActD causes mainly apoptotic cell death in different cell lines [16]. We investigated whether the cytotoxic effect of ActD in NMNAT1 KO cells was also apoptotic using Calcein-AM viability assays. Wild type and NMNAT1 KO U2OS cells were treated with ActD (40 nM) alone or in combination with the caspase-3 inhibitor (DEVD-fmk) or the necroptosis inhibitor (Nec1). DEVD-fmk significantly inhibited ActD toxicity in both wild type and NMNAT1 knockout cells, while Nec1 pretreatment did not cause significant changes in viability (Figure 3A). Apoptotic cell death was confirmed with high-content analysis. A kinetic caspase-3 activity assay was performed in the presence of DEVD-fmk (Figure 3B). ActD treatment caused a marked elevation in caspase-3 activity in both cell lines, which could be effectively inhibited by the caspase-3 inhibitor. In the NMNAT1 knockout cell line, caspase-3 activation was detected earlier, and the number of caspase positive cells was more than twice as high as in the wild type samples (Figure 3B,C). Cell necrosis was measured with lactate dehydrogenase (LDH) release 24 h after ActD treatment (Figure 3D). No significant change in LDH release was detected in WT cells. A significant release of LDH was detected in the NMNAT1 KO cell line.

### 2.3. The Relationship between Low Nuclear NAD^+^ and Cell Death

**Figure 4:**
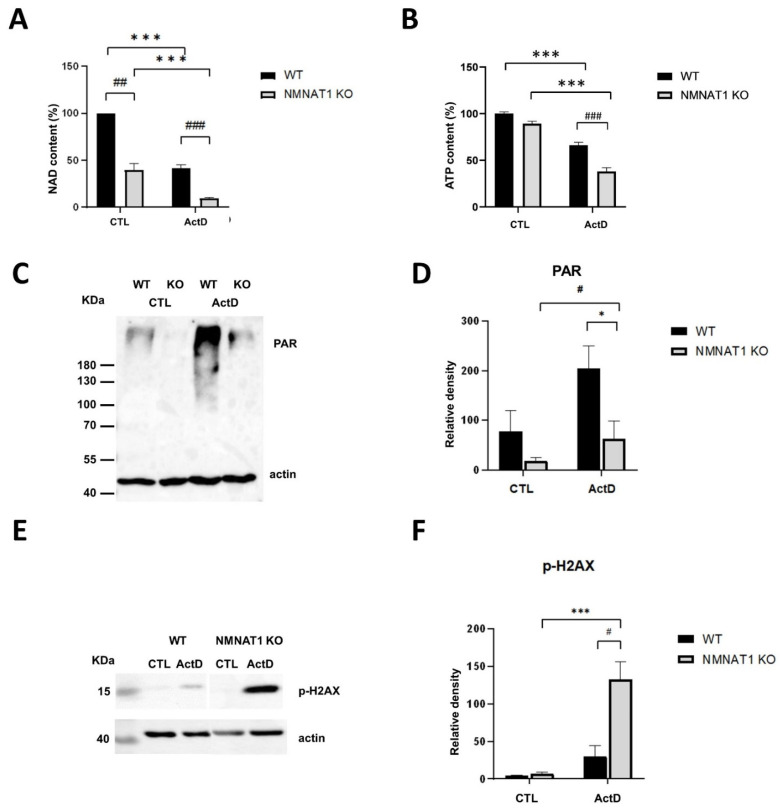
Low nuclear NAD^+^ inhibits PARylation-dependent DNA repair. Total NAD levels were determined from cell lysates of WT and NMNAT1 KO cells 24 h after actinomycin D (ActD) treatment and normalized to protein content (**A**). Bars marked with asterisks are significantly different from the control (Sidak’s test; *** *p* < 0.001, ## *p* < 0.01, ### *p* < 0.001). Bars marked with hash marks are significantly different from the corresponding treatment of the wild type cells (Sidak’s test; ## *p* < 0.01, ### *p* < 0.001). The ATP content was assayed 24 h after ActD treatment (**B**). Bars marked with asterisks are significantly different from the control (Dunnett’s test; *** *p* < 0.001, ### *p* < 0.001). Means ± SEM are shown on panels (**A**) (*n* = 4), (**B**) (*n* = 3). ActD-induced PARylation was detected by western blot 6 h after ActD treatment in WT and NMNAT1 KO U2OS cells (**C**). The relative density of PAR polymers is shown, normalized to beta-actin levels (**D**). Bars marked with hash marks are significantly different from the corresponding treatment of wild type cells (Sidak’s test; * *p* < 0.05, # *p* < 0.05). Phosphorylation of H2AX histone was detected by western blotting 6 h after ActD treatment in WT and NMNAT1 KO U2OS cells (**E**). The relative density of *p*-H2AX is shown, normalized to beta-actin levels (**F**). Bars marked with asterisks are significantly different from the control (Sidak’s test; *** *p* < 0.001, # *p* < 0.05). Bars marked with hash marks are significantly different from the corresponding treatment of wild type cells (Sidak’s test; # *p* < 0.05). The representative blots (**C**,**E**) and averages (±SEM) of the densities of three independent blots are shown (**D**,**F**).

We previously reported [4] that basal NAD^+^ levels are 60% lower in NMNAT1 KO cells compared to wild type U2OS cells. As ActD treatment induced significantly higher cytotoxicity in NMNAT1 KO cells, we measured the effects of ActD treatment on cellular NAD^+^ levels (Figure 4A). ActD caused a significant drop in NAD^+^ levels. In the KO cell line, NAD^+^ levels further dropped to 76% of the nontreated control, while a 60% decrease was observed in the treated wild type samples (Figure 4A). As ATP production is dependent on NAD^+^, we investigated whether ATP levels changed after ActD treatment (Figure 4B). As previously reported, the basal ATP levels were not significantly different between WT and KO cells [4]. ActD treatment caused a slight drop in ATP levels in wild type cells, while NMNAT1 KO cells showed a significant decrease in the total ATP level.

Low NAD^+^ levels inhibit the activity of nuclear NAD^+^-dependent enzymes, such as PARP1 or SIRT1 [17,18]. Actinomycin D causes DNA damage [19]. DNA damage activates the NAD^+^-dependent PARP1 enzyme, which interacts with NMNAT1 on PARP1-dependent promoters [20]. The two proteins may also interact at the sites of DNA damage.

PARylation was detected with western blot, using anti-poly (ADP-ribose) (anti-PAR) antibodies (Figure 4C). Excessive PARP activation was detected in wild type cells, 6 h after ActD treatment (Figure 4C,D), while KO cells displayed a significant decrease in PARP activation. DNA damage can be detected by measuring H2AX phosphorylation (p-H2AX or γH2AX). No significant DNA damage was detected by western blotting in the wild type U2OS cells (Figure 4E,F). In the KO cells, however, a high level of DNA damage could be seen 6 h after ActD treatment (Figure 4E,F). These data suggest that ActD-induced DNA damage fails to activate PARP1 in NMNAT1 KO cells and substrate deprivation of PARP1 limits the capacity of the cells to efficiently cope with DNA injury. Increased DNA damage may indicate impaired DNA repair.

**Figure 5:**
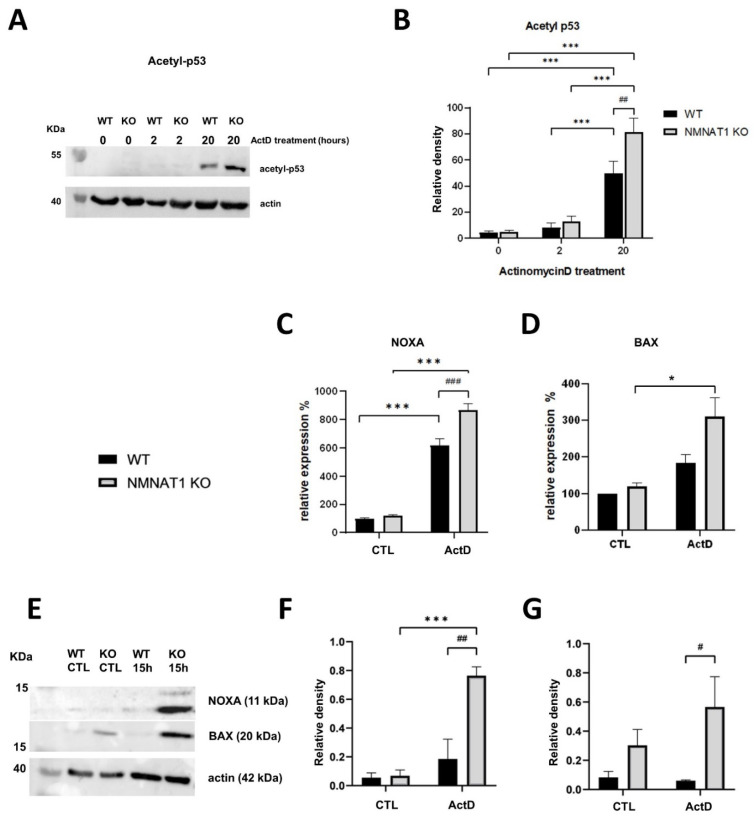
Low nuclear NAD^+^ activates SIRT1-dependent pro-apoptotic pathways. Acetylation of p53 protein was determined after 2 or 20 h of ActD treatment, using western blots (**A**). Acetyl-p53 levels were normalized to beta-actin levels. The average densitometries of three independent blots are shown (**B**). Bars marked with asterisks are significantly different from the control (Sidak’s test; *** *p* < 0.001). Bars marked with hash marks are significantly different from the corresponding treatment of the wild type cells (Sidak’s test; ## *p* < 0.01). Expression levels of pro-apoptotic NOXA (**C**) and BAX (**D**) genes were determined using the real-time quantitative PCR method. Cells were collected 15 h after actinomycin D (ActD) treatment. Bars marked with asterisks are significantly different from the control (Sidak’s test; * *p* < 0.05, *** *p* < 0.001). Bars marked with hash marks are significantly different from the corresponding treatment of the wild type cells (Sidak’s test; ### *p* < 0.001). The protein levels of NOXA and BAX genes were also determined using western blots. (**E**) Representative blots are shown. Relative densities of three independent blots of NOXA (**F**) or BAX (**G**) are shown, normalized to beta-actin levels. Bars marked with asterisks are significantly different from the control (Sidak’s test; *** *p* < 0.001). Bars marked with hash marks are significantly different from the corresponding treatment of the wild type cells (Sidak’s test; # *p* < 0.05, ## *p* < 0.01). Means ± SEM are shown on panels (**B**–**D**,**F**,**G**) (*n* = 3).

As SIRT1 is an NAD^+^-dependent deacetylase enzyme, acetylation of its target proteins (e.g., p53) [21] was expected to be elevated. Using western blotting, p53 acetylation on Lys382 was detected (Figure 5A). In the untreated samples, no acetylation was detected, and short (2 h) ActD treatment did not change p53 acetylation. However, after 20 h of ActD treatment, increased acetylation was detected in both WT and KO cells, showing a significantly higher level in the KO cells (Figure 5B). The increased acetylation of p53 may induce the expression of p53 dependent pro-apoptotic genes, such as *NOXA* or *BAX* [22]. Thus, the expression of these genes was determined at the mRNA (Figure 5C,D) and protein (Figure 5E–G) levels. Both NOXA (Figure 5C) and BAX (Figure 5D) mRNAs were elevated in both cell lines after 15 h of ActD treatment. Induction of the *NOXA* gene was significantly higher in the NMNAT1 KO cells (Figure 5C). Both NOXA (Figure 5E,F) and BAX (Figure 5E,G) proteins were significantly higher in the NMNAT1 KO cells compared to WT cells.

### 2.4. NMNAT1 Gene Inactivation Inhibits Cell Proliferation

**Figure 6:**
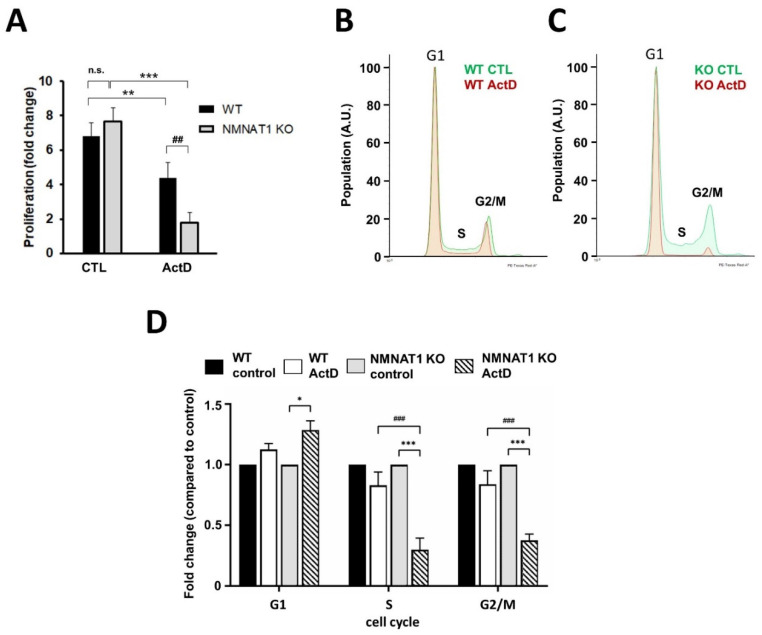
Actinomycin D causes decreased proliferation and cell cycle arrest on NMNAT1 KO U2OS cells. The proliferation of WT and NMNAT1 KO U2OS cells was measured using the high content analysis (HCA) method (**A**). The number of cells was determined on the day of treatment (day 1) and on day 5 (4 days after ActD treatment). The fold change of the cell number is plotted. Bars marked with asterisks are significantly different from the control samples on day 5 (Sidak’s test; *** *p* < 0.001, ** *p* < 0.01). Bars marked with hash marks are significantly different from the corresponding treatment of wild type cells (Sidak’s test; ## *p* < 0.01). The cell cycle was determined with propidium iodide (PI) staining and flow cytometry. Representative flow cytometry curves of wild type (**B**) and NMNAT1 KO (**C**) U2OS cells are shown. Quantitative analysis of the cell cycle phases is shown (**D**). Bars marked with asterisks are significantly different from the control samples (Tukey test; * *p* < 0.05, *** *p* < 0.001). Bars marked with hash marks are significantly different from the corresponding treatment of wild type cells (Tukey test; ### *p* < 0.001). Means ± SEM are shown on panels (**A**,**D**) (*n* = 3).

Cell proliferation was monitored in a high content analysis, determining the cell number before and four days after ActD treatment (Figure 6A). During the four-day period, the number of untreated WT and KO cells increased approximately seven-fold with no significant difference between the two cell lines. ActD treatment slowed down cell proliferation in both WT and KO cells, but KO cells showed a more dramatic decrease in proliferation compared to their wild type counterparts. Populations of cells at different cell cycle phases were determined with flow cytometry (Figure 6B–D). No significant differences were found between the two cell lines without treatment (Figure 6D). However, after ActD treatment, the ratio of populations at S and G2/M phases were markedly decreased in the NMNAT1 KO cell line (Figure 6C,D), while no significant change could be detected in the WT cells (Figure 6B,D).

**Figure 7:**
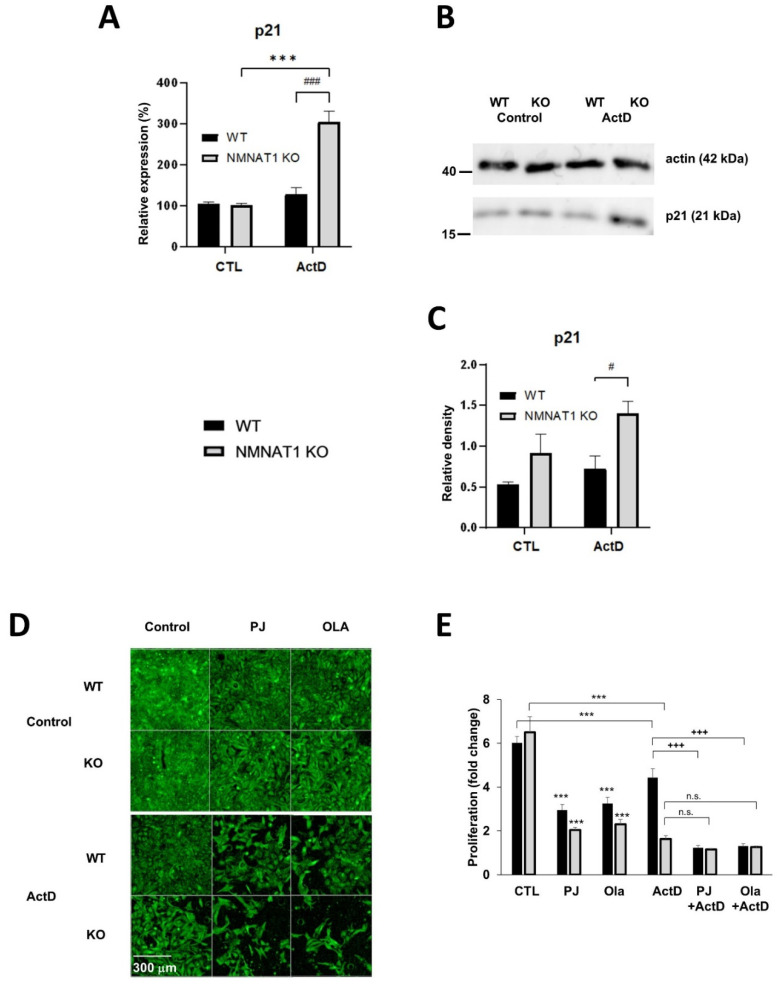
Mediators of Actinomycin D (ActD)-induced decreased proliferation. Expression levels of the p53-dependent gene p21 were determined with real-time quantitative PCR (**A**). Samples were collected 15 h after ActD treatment. Bars marked with asterisks are significantly different from the control (Sidak’s test; *** *p* < 0.001). Bars marked with hash marks are significantly different from the corresponding treatment of the wild type cells (Sidak’s test; ### *p* < 0.001). The protein levels of p21 were also determined using western blots. (**B**) A representative blot is shown. The relative density of p21 is shown, normalized to beta-actin levels (**C**). The average densities of three independent blots are shown. Bars marked with hash marks are significantly different from the corresponding treatment of the wild type cells (Sidak’s test; # *p* < 0.05). The proliferation of WT and NMNAT1 KO U2OS cells in the presence of ActD, PARP inhibitors, or their combination was measured using high content analysis (HCA) (**D**,**E**). The number of cells was determined on the day of treatment (day 1) and on day 5 (4 days after ActD treatment) and the fold change is plotted. Bars marked with asterisks are significantly different from the control samples from day 5 (Sidak’s test; *** *p* < 0.001). Bars marked with plus marks are significantly different from the ActD only treated samples on the same cell type (Sidak’s test; +++ *p* < 0.001, n.s.: not significant). Representative images taken on day 5 are shown in (**D**) and the averages (± SEM) of three independent experiments are shown in (**E**).

Acetylated p53 regulates cell proliferation through the induction of p21 gene expression [23]. ActD induced p21 mRNA (Figure 7A) and protein (Figure 7B,C) expression in the KO cells but not in the WT cells.

Since inhibition of PARP slows cell proliferation [24], we tested the effects of compromised PARylation on ActD-induced inhibition of cell proliferation in NMNAT1 KO cells (Figure 7D,E). PARP inhibitors decreased the proliferation rates in both lines. Combined treatment with ActD and PARP inhibitors significantly decreased proliferation in wild type cells, compared to cells treated with ActD only, but no further decrease could be detected in the NMNAT1 KO cells (Figure 7E).

### 2.5. Total RNA, Ribosomal RNA

**Figure 8:**
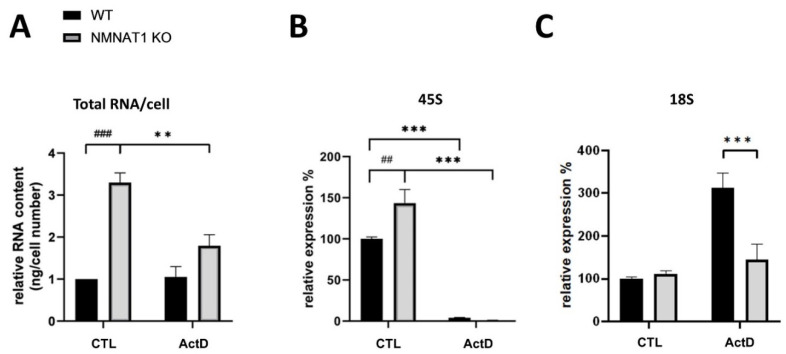
The effects of the NMNAT1 KO phenotype and Actinomycin D (ActD) treatment on RNA content. Relative RNA content was determined by counting the number of cells and measuring the total RNA content of the samples, after 24 h of ActD treatment. Results are expressed as fold changes compared to the wild type controls (**A**). Bars marked with asterisks are significantly different from the control (Sidak’s test; ** *p* < 0.01). Bars marked with hash marks are significantly different from the corresponding treatment of the wild type cells (Sidak’s test; ### *p* < 0.001). The amounts of 45S and 18S ribosomal RNAs were determined with real-time quantitative PCR (**B**,**C**). Bars marked with asterisks are significantly different from the control (Sidak’s test; *** *p* < 0.001). Bars marked with hash marks are significantly different from the corresponding treatment of the wild type cells (Sidak’s test; ## *p* < 0.01). Means ± SEM are shown on each panel (*n* = 3).

Both NMNAT1 [25] and ActD [26] affect RNA synthesis. To investigate RNA homeostasis in our model, the amount of total RNA (Figure 8A) and the expression of 45S (Figure 8B) and 18S (Figure 8C) ribosomal RNAs were measured. The NMNAT1 KO cells had 3× higher total RNA levels compared to their wild type counterparts (Figure 8A). ActD treatment significantly attenuated the elevation of total RNA in the NMNAT1 KO cells but did not affect WT cells (Figure 8A). The absence of NMNAT1 increased the expression of the pro-form of 18S ribosomal RNA (45S, Figure 8B). The expression of 45S RNA was completely blocked by ActD treatment in both cell lines. Interestingly, no differences in the amount of mature 18S rRNA (Figure 8C) between the two lines were detected. Surprisingly, ActD treatment elevated mature 18S rRNA levels in the wild type cells only (Figure 8C).

## 3. Discussion

Improving the efficiency of osteosarcoma therapy requires the development of new approaches. NAD^+^ is required for the rapid proliferation of cancer cells [27] and certain enzymes involved in NAD^+^ synthesis have already been identified as positive regulators of tumor progression. For example, overexpression of NAMPT contributes to cancer pathogenesis and development [28,29]. The p53-inducible protein, NMNAT2 [30], may also represent a therapeutic target in colorectal cancer [31]. SIRT3 activates NMNAT2 through deacetylation and increases NAD^+^ levels in non-small cell lung cancer cells [32]. However, knowledge about the role of NMNAT1 in tumors is limited. Our previous study [4] proved that NMNAT1 is required for DNA repair and survival of cisplatin and doxorubicin-treated osteosarcoma cells.

We performed a high-throughput cytotoxicity screening to identify novel pathways or compounds linked to the cancer-promoting role of NMNAT1. Our screen of 774 FDA-approved drugs identified nine compounds that showed an increased toxicity in the genetically edited U2OS osteosarcoma cells, which lack NMNAT1, compared to their wild type counterparts (Figure 1). With the exception of digoxin (Figure 2J), all hits were validated in follow-up experiments (Figure 2A–H). Of note, all validated hits are cancer chemotherapeutics. Five of them (daunorubicin, doxorubicin, idarubicin, epirubicin, and mitoxantrone) are anthracyclines and inhibit topoisomerase II. These findings confirm our previous results related to the protective role of NMNAT1 in doxorubicin-induced osteosarcoma cell death [4]. An interesting hit was the proteasome inhibitor, bortezomib, used for the treatment of hematologic malignancies (e.g., multiple myeloma, mantle cell lymphoma, and plasma cell leukemia) [33,34]. Proteasome inhibitors target processes related to tumor cell proliferation, cell death, and angiogenesis by modulating the expression of proteins, such as p53, Bcl-2, BAX, and NFκB pathway components [35]. Understanding the interrelationship between NMNAT1 and bortezomib requires further investigation.

ActD (also known as dactinomycin) had marked concentration-dependent toxic effects in NMNAT1 KO cells, even at low concentrations. We aimed to understand the mechanism behind the sensitizing effect of NMNAT1 on actinomycin D-treated osteosarcoma cells. ActD is a polypeptide [36] with bacteriostatic [37], HIV-suppressive [38], and anti-tumor [39] effects. ActD forms stable complexes with double-stranded DNA [26], leading to the inhibition of RNA synthesis [40]. ActD is used as an antitumor antibiotic to treat various malignant neoplasms, including Ewing’s sarcoma (a bone and soft tissue sarcoma) [41].

The mechanism by which ActD triggers cell death is poorly understood. ActD causes apoptotic cell death in osteosarcoma cells [16], but caspase-independent, non-apoptotic mechanisms have also been described in other cell types (e.g., in neuroblastoma cell lines) [39]. Our data using a caspase inhibitor and an RIP-1 targeted necroptosis inhibitor (necrostatin-1) suggest that apoptosis is the dominant form of cell death in ActD-treated osteosarcoma cells (Figure 3A–C), while the significance of necroptosis may be negligible (Figure 3A,D). Besides caspase activation, ActD triggered LDH release in the NMNAT1 KO cells. The LDH release was blocked with the caspase inhibitor and can be considered as a sign of secondary necrosis (Figure 3D).

Basal NAD^+^ levels in NMNAT1-deficient U2OS cells were 60% lower than in wild type cells, and ActD treatment further decreased NAD^+^ levels (Figure 4A). Therefore, we hypothesized that two major nuclear NAD^+^-dependent mechanisms underlie the tumor-promoting role of NMNAT1 in ActD-treated osteosarcoma cells: PARP and SIRT activities. PARP1 is an NAD^+^-dependent nuclear enzyme that is activated by DNA damage [42]. PARP1 is a major NAD^+^ consumer in cells exposed to genotoxic stimuli and poly (ADP-ribosyl)ation (PARylation) of nuclear proteins facilitates DNA repair [42]. We previously reported [4] that PARP1 activity is highly inhibited in cisplatin-treated NMNAT1 KO cells, resulting in enhanced DNA damage. Similarly, our current study revealed that ActD causes DNA damage and PARP activation. NMNAT1-deficient cells displayed restricted PARP activation and more severe DNA damage (Figure 4C–F). This finding supports our hypothesis that NMNAT1 KO sensitizes cells, at least in part, by compromising NAD^+^-dependent PARylation. The mechanism by which ActD causes DNA damage is poorly understood, but free radical-mediated DNA oxidation and topoisomerase inhibition may contribute to ActD-induced DNA breakage [43,44].

In addition to PARP1, the type III deacetylase, SIRT1, represents another NAD^+^-dependent nuclear enzyme [45]. The expression and activity of SIRT1 are upregulated in tumor cells [46] and SIRT1 expression is higher in drug-resistant cell lines and cancer patients undergoing chemotherapy [47]. Decreasing SIRT1 expression results in decreased cell proliferation and increased apoptotic cell death [48]. Thus, NMNAT1 deficiency may synergize with ActD, because SIRT1 activity is highly sensitive to the reduction in NAD^+^, leaving its targets acetylated. Indeed, acetylation levels of p53, one of the main substrates of SIRT1, could be detected 20 h after ActD treatment (Figure 5A,B). Furthermore, acetylation levels of p53 were significantly elevated in NMNAT1 KO cells compared to wild type cells (Figure 5A,B). This is in line with previous observations of higher p53 acetylation and apoptosis upon SIRT1 inhibition [49]. SIRT1 negatively regulates apoptosis [46] and SIRT1 upregulation is essential for cell survival after ActD treatment [49]. Consistent with the literature, we demonstrated increased mRNA and protein expression of the p53-dependent apoptosis mediators, NOXA and BAX [50], in the NMNAT1 KO cells (Figure 5).

The activation of p53 may also result in cell cycle arrest through the induction of p21 [50]. Induction of p21 was only detected in the ActD-treated NMNAT1 KO cells (Figure 7A–C). Elevated p21 expression was accompanied by decreased proliferation of ActD-treated NMNAT1 KO cells (Figure 6A). Moreover, cell cycle analysis revealed a significant decrease of cells in S and G2/M phases (Figure 6B–D), which is in line with the finding that ActD causes cell cycle arrest at the G1 phase [51]. These data suggest that the genetic inactivation of NMNAT1 negatively impacts cell cycle progression in a p53-dependent manner. Similar to our observations with cisplatin [4], the potent PARP inhibitors, PJ34 or olaparib, enhanced the anti-clonogenic effect of ActD in wild type cells but not in NMNAT1-deficient cells (Figure 7D,E).

An additional mechanism connecting NMNAT1 and ActD to p53 activation involves rRNA regulation. The p53 protein is linked to the oncoprotein MDM2, which mediates p53 ubiquitination and proteasome-dependent degradation [52]. This mechanism keeps nuclear p53 levels low. In the absence of ribosomal RNA, MDM2 interacts with ribosomal proteins, preventing the p53-MDM2 interaction. ActD is a ribosome biogenesis inhibitor [26]. NMNAT1 [25] also represses rRNA synthesis. Our results are in line with both findings (Figure 8B); we detected a three-fold elevation of total RNA levels in the NMNAT1 KO cells (Figure 8A). In cells with high basal levels of ribosomal biogenesis (e.g., NMNAT1 KO osteosarcoma cells, Figure 8C), blocking rRNA synthesis (e.g., by ActD) leaves more ribosomal proteins unbound and able to interact with MDM2. This results in the liberation of a high amount of p53, triggering apoptotic cell death. This may be an additional underlying mechanism for the increased apoptosis observed in the NMNAT1 KO cells. PARP inhibition also reduced matured 18S RNA in ActD-treated NMNAT1 KO cells, as PARP1 has a role in RNA biogenesis and metabolism [53,54]. While inhibition of PARP1 after γ-irradiation attenuates p53-dependent induction of p21 and MDM2 and suppression of G1 arrest [55,56], our results show that inhibiting both SIRT1 and PARP1 simultaneously by NAD^+^ depletion induces p21 expression and facilitates G1 arrest after ActD treatment.

In summary, our data identify NMNAT1 as a survival factor in ActD-treated osteosarcoma cells. The mechanism appears to be complex and may involve feeding nuclear enzymes with NAD^+^, including PARP1 for efficient DNA repair and SIRT1 for inhibition of p53-dependent cell death. Inhibition of either PARP1 [7,57] or SIRT1 [46] alone or in combination with chemotherapy has an advantage in eliminating tumors or inhibiting tumor invasion. Upon genotoxic stress, the slower NAD^+^ recycling limits both PARP1- and SIRT1-dependent cellular pathways, combining the benefits of inhibiting both pathways. Our results suggest that NMNAT1 inhibition and ActD may represent a novel therapeutic modality for the treatment of osteosarcoma. Once isoform-specific NMNAT1 inhibitors become available, the effects of these inhibitors in both osteosarcomas and other cancer types will be interesting.

## 4. Materials and Methods

### 4.1. Cell Culture

*Human* U2OS cells were grown in Dulbecco’s modified Eagle’s medium (DMEM, #12-604F, Lonza, Basel, Switzerland), containing 10% fetal bovine serum (#10500-064, GIBCO, Thermo Fisher, Waltham, MA, USA), L-glutamine, and penicillin-streptomycin, under standard cell culture conditions (humidified atmosphere, 37 °C, 5% CO_2_). The cells were regularly tested for mycoplasma contamination. NMNAT1 KO U2OS cells were generated with Crispr-cas9 technology, as reported in [4].

### 4.2. Cell Viability Assay for HTS Screening and Hit Validation

The Screen-Well^®^ FDA Approved Drug Library V2 (BML-2843-0100, Enzo Life Sciences, Farmingdale, NY, USA) was used for the screening. This compound library included 774 compounds approved by the US Food and Drug Administration (FDA).

Cells (2 × 10^4^ cells/well in 100 μL medium) were seeded into 96-well plates and grown for 24 h. The compounds of the library were transferred to the plates with a Tecan Freedom EVO liquid handling robot to a final concentration of 10 μM followed by a 24-h incubation. Then, the cells were stained by adding 50 μL of Calcein-AM (#17783, Sigma, St. Luois, MO, USA) solution at a final concentration of 1 μM. After incubating cells for 1 h at 37 °C, the fluorescent signal was measured with a Tecan Spark 20M (Tecan, Männedorf, Switzerland) multimode reader (Ex/Em = 485/530 nm). Viability was expressed as a percentage of the untreated control.

To validate the selected cytotoxic hits, cells (2 × 10^4^ cells/well in 100 μL medium) were seeded into 96-well plates and grown for 24 h. Hit compounds were purchased from different companies, as summarized in Supplementary Table S4. A concentration series was prepared to identify the lowest effective concentration of the compounds. After 24 h of treatment, Calcein-AM assays were used to determine cell viability, as described above.

### 4.3. Cell Proliferation Assay (High Content Analysis)

Cells (10^3^/100 μL/well) were seeded into Cell Carrier-96 ultra microplates (PerkinElmer, Waltham, MA, USA) and grown for 24 h. On the following day, ActD (1.25 nM; #S8964, Selleckchem, Houston, TX, USA) was added and the cells were incubated for 4 days. In the experiments involving PARP inhibitors, olaparib (S1060, Selleckchem, Houston, TX, USA, 10 μM) or PJ34 (S7300, Selleckchem, Houston, TX, USA, 10 μM) was added to cells 30 min before the ActD treatment. After 4 days, the plates were placed into an Opera Phenix High-Content Analyzer and counted using phase contrast images.

### 4.4. RNA Extraction, RNA Content and Quantitative PCR

The RNA Extraction and Quantitative PCR were performed as described in [4]. The RNA concentration was measured with a NanoDrop 1000 Spectrophotometer (Thermo Fisher, Waltham, MA, USA).

### 4.5. Caspase Activation and Lactate Dehydrogenase (LDH) Release Assay

Caspase activation and LDH Release were performed exactly as described in [4].

### 4.6. Western Blotting and Cellular NAD^+^/ATP Assays

Cellular NAD^+^ and ATP measurements and western blotting were performed as described in [4].

### 4.7. Cell Cycle Analysis by Flow Cytometry

Cells were synchronized by serum starvation. The cells were grown in T25 flasks (#90026, TPP, Trasadingen, Switzerland) for 1 day. When 60% cell confluence was reached, the cells were washed once with PBS and serum-free media was added. Cells were kept in the serum-free media for 24 h. Cells were then washed with PBS, trypsinized, and transferred into Falcon tubes. The cells were centrifuged at 300× *g* for 5 min, the supernatants were discarded, and the cells were resuspended in 3 mL of PBS. Then, the cells were centrifuged again (300× *g* for 5 min), the supernatant was discarded, and the cells were resuspended in 400 μL PBS. Ice-cold 70% ethanol (3 mL) was added slowly to the cells, and the samples were placed on ice for 30 min. Next, cells were centrifuged for 5 min at 300× *g*, the supernatant was discarded, and the cells were washed with 3 mL PBS. Finally, the supernatant was discarded and the cells were resuspended in 500 μL PBS. After adding 50 μL of 1 mg/mL RNAse A (#R4875, Sigma, St. Louis, MO, USA) solution (prepared in 0.1% Tween-20/5mM EDTA, heated in a 95 °C water bath for 30 min) and 5 μL of propidium iodide (PI; 1 mg/mL dH_2_O; #P4864, Sigma, St. Louis, MO, USA), the samples were incubated at room temperature for 1 h in the dark. The analysis was performed with a Novocyte 3000 (Acea Biosciences, Santa Clara, CA, USA) flow cytometer.

### 4.8. Statistical Analysis

Experiments were repeated at least three times and data are expressed as means ± SEM. GraphPad Prism 9 (La Jolla, CA, USA) was used for statistical analysis. The normal distribution of the data was determined by the D’Agostino-Pearson test. All the datasets showed normal distribution and were analyzed using two-way ANOVA followed by Sidak, Tukey, or Dunnett’s post-hoc tests.

## Data Availability

Primary data are available at: http://193.6.152.202:5000/sharing/MGX0oQjlV (accessed on 12 July 2021).

## Supplementary Materials

The following are available online at https://www.mdpi.com/article/10.3390/ijms22168869/s1.
