# Supplementary material for: NMNAT1 Is a Survival Factor in Actinomycin D-Induced Osteosarcoma Cell Death
Source: Int J Mol Sci. 2021 Aug 18;22(16):8869. doi: 10.3390/ijms22168869 (PMC8396190; doi:10.3390/ijms22168869)
Supplement: Supplementary file 1 [file ijms-22-08869-s001.zip › ijms-1318012-SI.pdf]

Table S1. Antibodies used for western blotting.

Antibody Specific for	Host Species	Cat#	Supplier	Dilution
poly(ADP-ribose)	mouse	N/A	produced in house (10H hybridoma)	1:500
NMNAT1	rabbit	sc-271557	Santacruz (Dallas, TX, USA)	1:1000
human actin	mouse	sc-47778	Santacruz (Dallas, TX, USA)	1:20000
BAX	rabbit	ab32503	Abcam (Cambridge, UK)	1:1000
NOXA	mouse	ab13654	Abcam (Cambridge, UK)	1:100
PUMA	rabbit	ab9643	Abcam (Cambridge, UK)	1:1000
p21	mouse	05-655	Sigma (St. Luois, MO,USA)	1:1000
anti-phospho histon H2AX	mouse	4418-APC-100	Trevigen (Gaithersburg, MD, USA)	1:1000
Acetyl-p53 (Lys379)	mouse	2570S	Cell Signaling (Danvers, MA, USA)	1:1000
anti-mouse IgG (HRP)	horse	7076S	Cell Signaling (Danvers, MA, USA)	1:3000
anti rabbit IgG (HRP)	goat	7074S	Cell Signaling (Danvers, MA, USA)	1:1500

Table S2. Antibodies and dyes used for High Content Analysis.

Antibody Specific for	Host Species	Cat#	Supplier	Dilution/Concentration
CellEvent™ Caspase-3/7 Green Detection Reagent	-	C10423	Thermo Fischer (Waltham, MA USA)	7 μM

Table S3. Sequences of primers, used in quantitative PCR experiments.

Primers	Fw	Rw
hNMNAT1	*AAAGGCCTGGAAGGAAGAGG*	*CCCATAGTTGGCCACGATTT*
hNMNAT2	*CATATTTCCTGGTGGCATCT*	*CTGGTCCTGCCTATGTGGTT*
hNMNAT3	*GCGCACATCCAGGAAATAGT*	*TGGCACTGATCTCATTCTGC*
hBAX	*CTGCAGAGGATGATTGCCG*	*TGCCACTCGGAAAAAGACCT*

hNOXA	*CTGGAAGTCGAGTGTGCTACTC*	*TGAAGGAGTCCCCTCATGCAAG*
h45S	*ACCCACCCTCGGTGSGA*	*CAAGGCACGCCTCTCAGAT*
h18S_45S	*CTCACCACGGGAAACCTCAC*	*CGCTCCACCAACTAAGAACG*
hTIGAR	*ATGAGGACAAAGCAGACCATGC*	*GCTTTGCCTTCTACAACCCC*
hPUMA	*GAGCAGGGCAGGAAGTAACAA*	*GGCAGACCCCATGCCAAAT*
hp21	*AGTCAGTTCCTTGTGGAGCC*	*CATTAGCGCATCACAGTCGC*
h36B4	*CCATTGAAATCCTGAGTGATGTG*	*GTCGAACACCTGCTGGATGAC*
hcyclophilin	*GTCTCCTTTGAGCTGTTTGCAGAC*	*CTTGCCACCAGTGCCATTATG*

Table S4. Compounds which have been used for the treatments

Compound	Cat#	Supplier
Z-DEVD-fmk	S7312	Selleckchem<br>(Houston, TX, USA)
Necrostatin 1	480065	Calbiochem<br>(Billerica, MA, USA)
Actinomycin D	S8964	Selleckchem<br>(Houston, TX, USA)
Daunorubicin	D8809	Sigma (St. Luois, MO,USA)
Bortemozib	HY-10227/CS-1039	MedChemExpress<br>(New Jersey, NJ, USA)
Digoxin	D6003	Sigma<br>(St. Luois, MO,USA)
Doxorubicin	OGYI-T-8808/04	Teva<br>(Debrecen, Hungary)
Mitoxantron-+dihyrochlorid	M6545	Sigma<br>(St. Luois, MO,USA)
Teniposide	1787	Selleckchem<br>Houston, TX, USA)
Idarubicin	S1228	Selleckchem<br>(Houston, TX, USA)
Epirubicin	S1223	Selleckchem<br>(Houston, TX, USA)
RNAse	R4875	Sigma<br>(St. Luois, MO,USA)
Propidium iodide	P4864	Sigma<br>(St. Luois, MO,USA)
